# Carbene-based Difluoromethylation of Bisphenols: Application to the Instantaneous Tagging of Bisphenol A in Spiked Soil for Its Detection and Identification by Electron Ionization Gas Chromatography-Mass Spectrometry

**DOI:** 10.1038/s41598-019-53735-9

**Published:** 2019-11-22

**Authors:** Carlos A. Valdez, Roald N. Leif, Saphon Hok

**Affiliations:** 1 Physical and Life Sciences Directorate, Lawrence Livermore National Laboratory, Livermore, CA 94550 USA; 2 Nuclear and Chemical Sciences Division, Lawrence Livermore National Laboratory, Livermore, CA 94550 USA; 3 Forensic Science Center, Lawrence Livermore National Laboratory, Livermore, CA 94550 USA

**Keywords:** Environmental sciences, Chemistry

## Abstract

The rapid and efficient difluoromethylation of a panel of eleven bisphenols (BPs) for their enhanced detection and identification by Electron-Ionization Gas Chromatography-Mass Spectrometry (EI-GC-MS) is presented. The derivatization employs the inexpensive, environmentally benign agent diethyl (bromodifluoromethyl) phosphonate (DBDFP) as a difluorocarbene-generating species that converts the BPs into *bis*-difluoromethylated ethers that can be detected and identified by GC-MS means. Key attributes of the protocol include its extreme rapidity (30 seconds) at ambient temperature, high specificity for BPs amidst other alcohol-containing analytes, and its biphasic nature that allows for its convenient adaptation to the analysis of BPs in organic as well as aqueous matrices. The protocol furnishes stable, novel BP ethers armed with a total of four fluorine atoms for their subsequent analysis by EI-GC-MS. Furthermore, each derivatized bisphenol exhibits unique retention times vastly different from their native counterparts leading to their unequivocal identification. The effectiveness and robustness of the developed methodology was applied to the tagging of the most famous member of this family of compounds, bisphenol-A (BPA), when spiked (at 1 μg.g^−1^ concentration) in the physically and compositionally complex Nebraska EPA standard soil. The method detection limit (MDL) for the *bis*-difluoromethylated BPA was determined to be 0.01 μg.mL^−1^. The *bis*-difluoromethylated BPA was conveniently detected on the organic layers from the biphasic, derivatized mixtures, highlighting the protocol’s practicality and utility in the rapid, qualitative detection of this endocrine disruptor during environmental analysis.

## Introduction

Among the most persistent chemicals in the environment and whose emerging negative reputation is starting to garner considerable supporting data are the bisphenols. Bisphenols (BPs) are a class of aromatic hydrocarbons comprising two phenolic units linked together by a carbon atom (or a heteroatom such as sulfur) that may be, in the simplest member of the family, a methylene unit (CH_2_) or a more elaborate structural motif (*e.g*. methylphenyl, *bis*-trifluoromethyl). While the two phenolic units may be linked symmetrically in three different ways, it is the one resulting in the 4,4′-bisphenol geometric isomer that has become the most employed in the chemical industry. Due to their chemical make-up and disposition for various chemical modifications at their hydroxyl moieties, bisphenols have played an important role in numerous industrial processes such as the manufacture of plastics (*e.g*. polycarbonate), various food packaging materials, dental sealants, flame-retardants and epoxy resins^1,2^. As it can be anticipated from their involvement in these applications, their eventual disposal has resulted in their dissemination in waste streams that eventually lead into various parts of the environment (*e.g*. soils, waterways, lakes). It is their indifference towards nature-based chemical modifications (*e.g*. hydrolysis, oxidation) that bestows in them the ability to remain in the environment and eventually find enough soil motility to be found basically anywhere. However, a far more alarming concern lies in their recognized toxicity and their high-ranking status in the constantly expanding list of endocrine disrupting compounds (EDCs)^3–6^. Over the last few years, numerous studies directly linking BPs to irreparable damage to neurological and hormonal systems in vertebrates and invertebrates have appeared^7,8^. Aside from their recognized toxicity and their environmental persistence, their ubiquitous existence in virtually every tested individual has certainly served to critically heighten public concern on these compounds^9–12^. Currently, research programs are being spearheaded by various research agencies to find alternative materials to replace bisphenols in the industrial manufacturing process and reduce its environmental and biological prevalence in society^3,13^. However, it is well recognized that this represents a long-term aim for various reasons, one of the most important ones being the fact that new alternatives for these crucial chemical building blocks in industry must be found and evaluated not only for their performance relative to bisphenols, but for their potentially toxic profile as well.

As a result of the cumulative literature demonstrating their toxicity and environmental persistence, analytical methods for their effective detection have been developed over the years. These methods have relied heavily on their intrinsic UV-absorption profiles resulting in the development of numerous LC-MS-based techniques^14–16^. Within the ambit of GC-MS analysis, traditional derivatization methods ranging from silylation to acylation to alkylation in order to produce derivatives with enhanced volatility than their parent compounds have been successfully employed for their analysis^17–19^. These derivatization methods have played important roles in BP extraction techniques from various matrices including soils. Out of all these, silylation (using BSTFA) stands out as one of the most used ones showcasing recoveries of BPA from agricultural/industrial soils and even sewage sludge with very high ranging values bordering almost complete recovery (~90 to >100%)^20,21^. However, these analytical procedures involve time-consuming pre-concentration, derivatization and post-concentration steps in addition to the non-specificity of the derivatization step resulting in the chemical modification of other analytes as well as interferences present in the matrix.

The work presented herein has its origins in the goal of providing an additional derivatization method tailored specifically for acidic, phenol-containing compounds. The method is rooted in the use of the difluoromethyl (CF_2_H) moiety, an isosteric equivalent to the methyl group (CH_3_), as a tag for the phenolic oxygens in bisphenols leading to the formation of new derivatives that bear a total of four fluorine atoms. An attractive feature of the protocol, and one that is not shared with the current protocols for bisphenol analysis, is the lack of an extraction step during its execution, thus leading to the rapid derivatization of the bisphenol in a given matrix. In the following protocol the extraction and derivatization steps take place simultaneously as time progresses (i.e. *in situ* derivatization). Thus, it is this anticipated that the process will furnish ether derivatives not only with increased volatility but enhanced sensitivity towards detection by mass spectral means as a result of the electron capturing ability of the added fluorine atoms^22,23^.

## Results and Discussion

**Figure 1:**
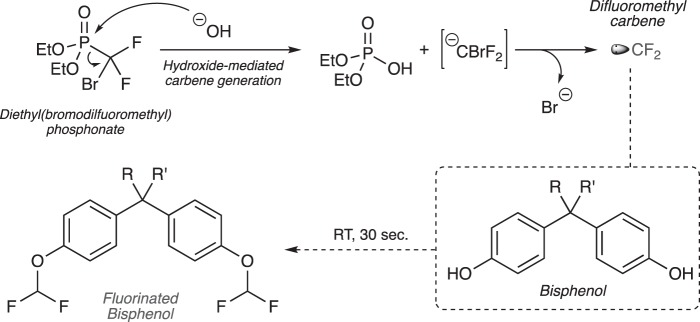
Overall derivatization strategy for bisphenols described in this work using DBDFP at room temperature.

Use of the fluorinated carbon units, mostly as the difluoromethyl (-CF_2_H) and the trifluoromethyl (-CF_3_) moieties, as isosteric alternatives for the methyl (-CH_3_) moiety in drug discovery is well documented^24^. Their use over the methyl group stems for the fact that once nitrogens, sulfurs and oxygen atoms present in a drug candidate are modified with this functionality, the net result is the blockade of metabolizing enzymes at that position that results in the increase of a drug’s bioavailability and serum half-life. Equally important and within the realm of analytical chemistry, fluorine-bearing substituents play a crucial role as valuable chemical tags for analytes whose conventional detection by GC-MS is problematic as a result of their inherent low ionization potential^25^. Introduction of fluorine atoms into analytes, mostly accomplished as the universally-employed trifluoroacetyl or pentafluorobenzyl groups, act in synergistic and additive fashion to enhance their detection by augmenting their electron-capture ability. Introduction of the trifluoroacetyl moiety is accomplished using trifluoroacetic anhydride (TFAA) while the pentafluorobenzyl tag requires the use of pentafluorobenzyl bromide in the presence of a base^26,27^. As efficient and established as these reactions are in the analytical chemistry toolbox of derivatization methods, the cross-reactivity of the reagents with virtually any nucleophile is unavoidable leading to the fluorination of most, if not all, analytes in a mixture. Even though the areas of complex data analysis, processing and deconvolution have experienced an exponential evolution in the last decade, the avoidance of unnecessary, derivatized side-products that can interfere with the analysis has remained a prime and often welcomed requirement in analytical chemistry. Now, with regards to the difluoromethyl group, clever ways have been devised by chemists to introduce them into molecules, with the majority of these applicable for only modifying acidic hydroxyl groups (*i.e*. phenols)^28–30^. Most of these methods involved heating (>100 °C) for prolonged periods of time (often >16 h), until the Zafrani group introduced the stable and environmentally benign agent diethyl (bromodifluoromethyl) phosphonate (DBDFP) that expedited the difluoromethylation of acidic phenols under much milder conditions^31^. Due to its exclusive employment in the fields of medicinal chemistry and drug development, the difluoromethyl tag has experienced extremely limited use in the fields of analytical chemistry and forensic science. Earlier work by our group focused on the use of DBDFP for the derivatization of chlorinated phenols (*p*K_*a*_ ~ 7–9) and demonstrated its efficiency, speed, and ability for detection of particularly toxic species by Proton and Fluorine Nuclear Magnetic Resonance (^1^H and ^19^F-NMR) spectroscopy^29^. Building on this work, we turned to expanding the methodology for the derivatization of bisphenols and their subsequent analysis by GC-MS means as shown diagrammatically in Fig. 1. Thus, under the basic conditions used in the protocol, DBDFP breaks down into diethylphosphonic acid and a fleeting bromodifluoromethyl carbanion that undergoes the loss of a bromide ion to generate the highly reactive difluorocarbene species that immediately reacts with the bisphenol to furnish the *bis*-difluorinated product (Fig. 1).

At the outset, we sought to determine the number of equivalents needed to successfully derivatize a given bisphenol without generating too many by-products from the degradation of excess reagent. After determining that 1.5 equivalents per hydroxyl group (thus a total of 3 for all bisphenols in this study) were sufficient for the derivatization of BPA without the creation of many by-products, we performed the derivatization on ten additional, structurally diverse bisphenols (Table 1). Interestingly, we were delighted to find that using large excess of DBDFP did not deleteriously affect the course of the reaction or the subsequent data analysis by providing additional by-products or interferences. Naturally, when encountering real world samples, one makes use of excess derivatization reagents to make sure that all of the analytes have been modified (*e.g*. BSTFA-mediated silylation), which is the reason why we employ such elevated concentrations when dealing with the soil samples (*vide infra*). Two interesting points that arise from the GC-MS studies of the reaction are, 1) the derivatization results in fluorinated bisphenol products with shorter retention times relative to the parent bisphenol, thus making them particularly useful in the analysis of late eluting analytes (*e.g*. B-FL with 38.7 min, SI, Page S16) with the difference in retention times between the derivatized and underivatized bisphenols ranging from 3.6–5.9 min., and 2) the protocol yields products that are *m/z* + 100 relative to the starting bisphenols, thus providing a strong, diagnostic, molecular ion peak when analyzing the data (Table 1).

**Table 1:** Gas chromatographic properties for the native and *bis*-difluoromethylated bisphenols described in this work.

BP	Structure	MW	MS peaks	RT (min.)^*a*^	BP Product	MW	MS peaks	RT (min.)^*b*^	ΔRT^*c*^
G		312	297 (999), 298 (405), *312 (267)*, 141 (130), 43 (114)	30.1		412	397 (999), 398 (221), *412 (128)*, 43 (97)	26.4	3.7
E		214	199 (999), *214 (281)*, 200 (157), 91 (80), 77 (74)	27.2		314	299 (999), *314 (253)*, 249 (176), 300 (128), 247 (78)	23.4	3.8
C		256	241 (999), *256 (261)*, 242 (217), 133 (212), 77 (98)	28.9		356	341 (999), 342 (180), *356 (177)*, 133 (93), 183 (85)	25.3	3.6
F		200	107 (999), *200 (915)*, 199 (393), 77 (214), 94 (189)	26.5		300	233 (999), *300 (703)*, 165 (267), 107 (197), 183 (172), 51 (169)	22.8	3.7
AF		336	267 (999), *336 (480)*, 199 (246), 197 (220), 99 (195), 268 (168), 169 (120)	26.2		436	367 (999), *436 (505)*, 317 (225), 368 (149), 249 (127), 201 (119), 200 (116), 299 (110)	21.0	5.2
AP		290	275 (999), 276 (236), 181 (132), *290 (109)*, 152 (93)	33.6		390	375 (999), 376 (215), 181 (86), 247 (58), *390 (46)*, 313 (45)	29.7	3.9
BP		352	275 (999), *352 (248)*, 181 (226), 259 (214), 276 (214), 165 (138)	37.7		452	375 (999), 309 (375), *452 (243)*, 376 (223), 181 (212), 165 (153)	33.7	4.0
FL		350	*350 (999)*, 257 (506), 351 (273), 349 (220), 226 (166), 255 (111), 258 (108)	38.7		450	*450 (999)*, 307 (511), 257 (475), 383 (445), 451 (306), 226 (260), 239 (122)	34.8	3.9
Z		268	225 (999), *268 (716)*, 131 (379), 107 (370), 199 (339), 197 (180)	32.3		368	325 (999), *368 (695)*, 157 (668), 301 (491), 299 (458), 107 (408), 181 (406)	28.5	3.8
S		250^*d*^	*250 (999)*, 110 (653), 141 (502), 65 (182), 142 (397)	33.3		350	191 (999), 141 (733), 109 (423), *350 (259)*, 95 (252)	27.4	5.9
A		228	213 (999), *228 (299)*, 214 (184), 119 (182), 91 (96)	27.8		328	313 (999), 119 (249), 314 (245), 169 (210), *328 (173)*	24.0	3.8

The corresponding masses for the more abundant fragment ions along with their relative intensity to the base peak have been in included (in parentheses). In the MS peaks columns, the molecular ion peaks for each compound have been italicized.

^*a*^Retention time of native bisphenol. ^*b*^Retention time of *bis*-difluoromethylated bisphenol. ^*c*^ΔRT = RT_(BP)_ − RT_(Fluorinated BP)_. ^*d*^Low response under GC conditions employed.

**Figure 2:**
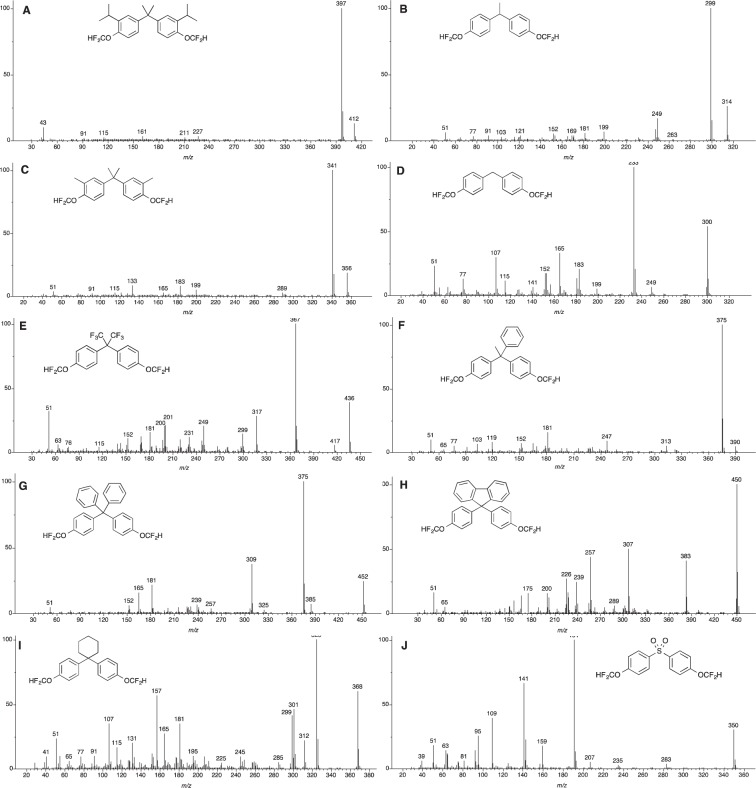
Mass spectra for all derivatized bisphenols, as their *bis*-difluoromethylated ethers, studied in this work.

In all the derivatized bisphenols studied in this work, the molecular ion peak is highly visible and in the case of bisphenol FL it is the base peak in the mass spectrum. In all other cases studied, the base peak was found to correspond to the species generated from the cleavage of one of the C-C bonds at the bridging carbon of the *bis*-difluoromethylated product (Fig. 2). This mode of ionization can be anticipated as the resulting radical carbocation generated at the bridging carbon is a highly stabilized doubly benzylic species while it is an even more stable trityl species where the remaining R group attached to the bridging carbon is a phenyl group. Thus, starting with *bis*-difluoromethyl bisphenol G (BP-G(CF_2_H)_2_), its relatively simple mass spectrum shows the molecular ion peak at *m/z* = 412 [M]^+^ as well as the spectra’s base peak at *m/z* = 397 [M − CH_3_]^+^ (Figs. 2A and SI, Page S19). Similarly, in the case of BP-E(CF_2_H)_2_, one can observe its molecular ion peak at *m/z* = 314 [M]^+^ while the base peak can be clearly noted at *m/z* = 299 [M − CH_3_]^+^ (Figs. 2B and SI, Page S13) arising from the loss of the bridging carbon’s methyl group. Interestingly, it is the loss of a methyl rather than a proton to yield a more stable tertiary radical carbocation species that provides the base peak in the spectra of BP-E(CF_2_H)_2_, a dominant fragmentation pattern that is also exhibited by the native BPE (SI, Page S12). Other fragments that can be accounted for but yield very low abundance are the ones at *m/z* = 263 [M − CF_2_]^+^ and *m/z* = 247 [M − OCF_2_]^+^. For the tetramethylated bisphenol analog, compound BP-C(CF_2_H)_2_, its mass spectrum seems to also offer very few fragment ions that include the molecular ion peak at *m/z* = 356 [M]^+^ and its base peak at *m/z* = 341 [M − CH_3_]^+^ arising from the loss of one of the methyl groups at the bridging carbon (Figs. 2C and SI, Page S11). Other lower abundance peaks that can be observed appear at *m/z* = 199 [M − C_7_H_5_OF_2_]^+^ arising from the loss of one of the difluoromethoxyphenyl arms from the bridging carbon, and *m/z* = 289 [M − OCF_2_]^+^ this last one exhibiting very low abundance. The derivative of the simplest bisphenol analyzed in the study, BP-F(CF_2_H)_2_ incidentally provides a complex mass spectrum (Figs. 2D and SI, Page S15). In the spectrum, one can observe a strong molecular ion peak at *m/z* = 300 [M]^+^ and the base peak at *m/z* = 233 [M − OCF_2_H]^+^ that is attributed to the loss of one of the difluoromethoxy groups from one of the phenyl rings. Another less intense peak in the mass spectrum that can be accounted for with structural loss from the molecule is *m/z* = 249 [M − CF_2_H]^+^.

For the bisphenol featuring two trifluoromethyl groups at the bridging carbon, BP-AF(CF_2_H)_2_, the mass spectra clearly shows the molecular ion peak at *m/z* = 436 [M]^+^ as in its underivatized form (*m/z* = 336 [M]^+^) (Figs. 2E and SI, Page S4). The base peak for BP-AF(CF_2_H)_2_ was found at *m/z* = 367 [M − CF_3_]^+^ leading to the tertiary, trifluoromethyldibenzyl radical cation (SI, Page S5). In reference to the bisphenols featuring a phenyl ring as one of the substituents at the bridging carbon, we begin our discussion with BP-AP(CF_2_H)_2_ which provides us with one of the simplest mass spectra in our study (Figs. 2F and SI, Page S7). The spectrum is dominated by the presence of a low molecular ion peak at *m/z* = 390 [M]^+^ and the base peak at *m/z* = 375 [M − CH_3_]^+^ arising from loss of the methyl group at the bridgehead, leaving a stabilized trityl radical cation. In the case of BP-BP(CF_2_H)_2_ one can observe the molecular ion peak at *m/z* = 452 [M]^+^ and the base peak at *m/z* = 375 [M − Ph]^+^ arising from loss of one of the phenyl rings at the bridgehead for a species that resembles the one obtained with BP-AP(CF_2_H)_2_ (*vide supra*) (Figs. 2G and SI, Page S9). Other peaks of interest that are significant in the spectrum are *m/z* = 309 [M − C_7_H_5_OF_2_]^+^ arising from the loss of one of the substituted phenyl groups to generate a trityl radical carbocation but not as strong as the one featured on the base peak.

**Figure 3:**
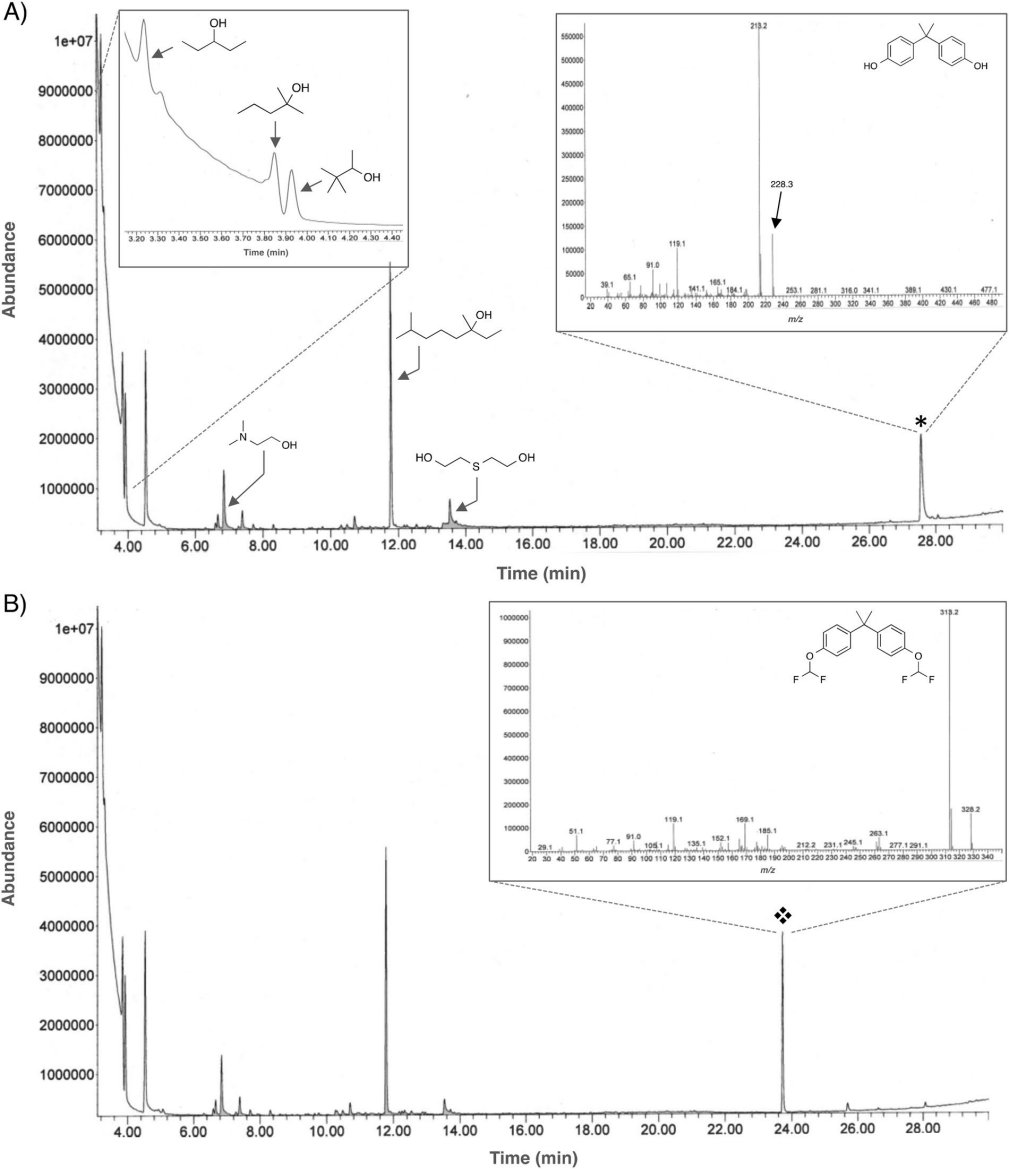
EI-GC-MS chromatographs showing the before (**A**) and after (**B**) difluoromethylation of BPA in the presence of other alcohols. The shift in retention time of the signal for the native BPA (*) as it gets converted to its fluorinated derivative (❖) while the other alcohols remain unmodified can be clearly observed.

One interesting product that provides a rich mass spectrum as its fluorinated derivative is BP-FL(CF_2_H)_2_. In the spectrum one can see several strong peaks arising from the breakdown of the product in and something quite interesting, the base ion peak is also the molecular ion peak at *m/z* = 450 [M]^+^. Other peaks in the spectra include one at *m/z* = 383 [M − OCF_2_H]^+^ representing the loss of one of the difluoromethoxy groups. The peak at *m/z* = 307 [M − C_7_H_5_OF_2_]^+^ represents the loss of one of the substituted phenyl rings from the bridgehead junction to yield a stable trityl radical carbocation akin to the process observed for BP-BP(CF_2_H)_2_ (Figs. 2H and SI, Page S17). In similar fashion, the spectrum of BP-Z(CF_2_H)_2_ provides us ion fragments that seem to be analogous to the spiro analogs like BP-FL(CF_2_H)_2_. Although not possessing its molecular ion peak at *m/z* = 368 [M]^+^, it features a base peak at *m/z* = 325 [M – C_3_H_7_]^+^ representing a complex fragmentation at the cyclohexyl spiro moiety (Figs. 2I and SI, Page S23). Another diagnostic peak for this compound is the one at *m/z* = 301 [M − OCF_2_H]^+^ representing the loss of one of the difluoromethoxy groups. One of the most important bisphenols studied in this work is BPS due to its long time notoriety as a suitable substitute of BPA for manufacturing processes but still remaining a threat to human health as evidenced by numerous studies^32^. During the course of our studies, the native BPS possessed low response during the EI-GC-MS analysis (SI, Page S20) a characteristic that was diametrically obvious in its derivatized counterpart BP-S(CF_2_H)_2_. In the derivatized compound, BP-S(CF_2_H)_2_, one can observe the molecular ion peak at *m/z* = 350 [M]^+^ and the base peak at *m/z* = 191 (Figs. 2J and SI, Page S21). Other peaks include *m/z* = 283 [M − OCF_2_H]^+^ representing the loss of one of the difluoromethoxy groups. Last but certainly not the least is BPA analog (BPA(CF_2_H)_2_) that features a fairly strong and clear molecular ion peak at *m/z* = 328 [M]^+^ arising and the base peak at *m/z* = 313 [M − CH_3_]^+^ originating from the loss of one of methyl groups at the bridging carbon (Inset of Figs. 3B and SI, Page S25).

As the protocol involves a highly basic solution for the generation of the difluorocarbene species, we hypothesized that the acidic bisphenols would be amenable for selective derivatization over other less acidic alcohol species. Thus, in order to test the selectivity of the protocol for bisphenols over other alcohols, we carried out the difluoromethylation of BPA in the presence of six other alcohols. The alcohols were chosen so as to span a range of structural diversity and reactivity. Thus, incubation of BPA spiked in a mixture of 3-pentanol, 2-methyl-2-pentanol, 2,2-thiodiethanol, pinacolyl alcohol, 3,7-dimethyl-3-octanol and *N,N*-dimethylaminoethanol followed by treatment of the mixture with excess DBDFP resulted in the sole derivatization of BPA over the other alcohols (Fig. 3). This demonstrates that the protocol is a viable method for the specific labeling of other acidic alcohols (*i.e*. chlorophenols, resorcinols) under the basic conditions employed for its execution^23^. The observed selectivity lies in the magnitude of the *p*K_a_ values for phenols that are orders of magnitude lower (*i.e*. ~7–8) than those exhibited by most alcohols (*i.e*. ~15–17). Consequently, under the basic conditions (pH ~ 12.8) at which the derivatization is conducted, the phenoxide not the alkoxide anion is the predominant species in the mixture and thus the most reactive towards difluoromethylation. Using the Henderson-Hasselbalch equation (Eq. 1) one can deduce that at pH = 12.8, the bisphenols practically exist as their phenoxide ions (with a *p*K_a_ = 7, 99.9%) while the aliphatic alcohols employed in this study would only marginally exist as their alkoxide counterparts (with a *p*K_a_ = 16, 0.001%).

1 $${\rm{pH}}=p{{\rm{K}}}_{{\rm{a}}}+\,\log (\frac{[{{\rm{A}}}^{-}]}{[{\rm{HA}}]})$$

In addition, one can invoke the much higher nucleophilicity of a phenoxide anion over that of an alkoxide anion as a reason for the selectivity observed in the process, however, this may not be a relevant contributor to the observed behavior as excess of the DBDFP is employed as eventually that could lead to the derivatization of the other alcohols.

**Figure 4:**
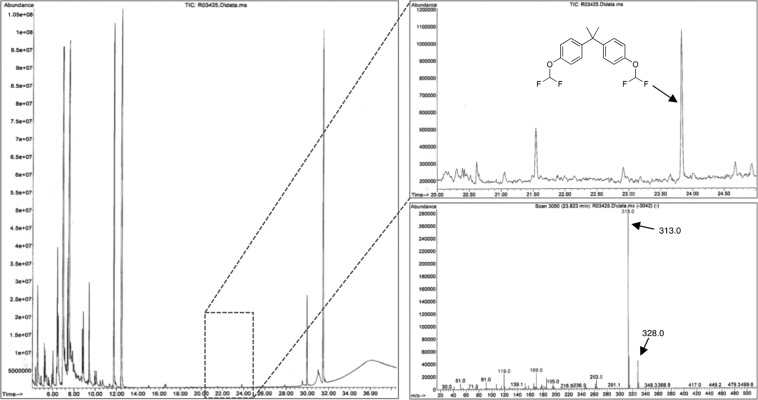
Difluoromethylation of BPA present at a 10 μg.g^−1^ concentration in Nebraska soil.

**Figure 5:**
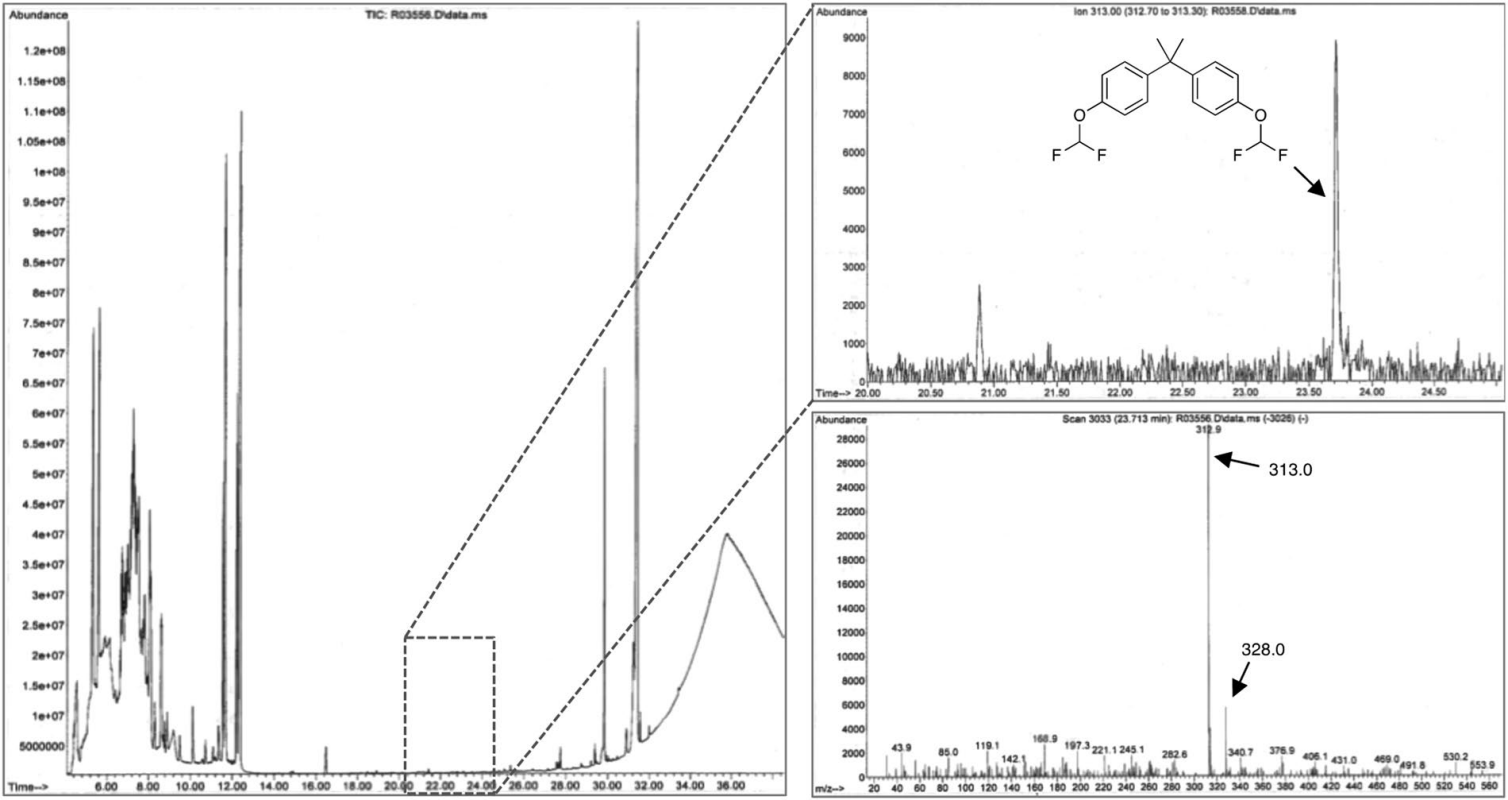
Difluoromethylation of BPA present at a 1 μg.g^-1^ concentration in Nebraska soil.

In order to exploit the advantage that this protocol offers over other derivatization methods, we decided to test its derivatizing power in a Nebraska soil sample that was spiked with BPA initially at a 10 μg.g^−1^ concentration and then at a 1 μg.g^−1^ concentration. The protocol involves the direct conversion of the contaminated soil into a biphasic mixture (KOH/H_2_O//CH_3_CN) followed by the *in situ* derivatization of BPA with DBDFP. Upon vortexing and subsequently allowing the soil residue to settle to the bottom of the vial along with the aqueous layer, the top acetonitrile layer was then aliquoted, dried and analyzed by GC-MS. Nebraska soil was selected as a matrix due to its composition that encompasses several characteristics not normally present in other soils such as a total organic content (TOC, 1.9%) that provides several organic analytes that can act as interferences. The soil also possesses a large percentage of silt (58%) and clay matter (32%) that are recognized as challenging matrices in the field of soil extraction and analysis due to their inherent reactivity and its fine particulate texture making it difficult for derivatizing agents to access trapped analytes^33^. When carrying out the derivatization on the Nebraska soil, we were delighted to find it was successful at tagging the BPA providing its *bis*-difluoromethylated ether in enough concentration to be easily detected by GC-MS means (Fig. 4). After this initial assessment at 10 μg.g^−1^ BPA concentrations, we decided to explore the derivatization at an order of magnitude lower concentration of BPA (1 μg g^−1^) in the same soil. Again, the derivatization of BPA was successful in yielding the *bis*-difluoromethylated analog in the soil. Interestingly, when dealing with such a low concentration of the analyte, we made use of a selected-ion extraction mode (*m/z* = 313, strongest ion in the chromatograph) in order to unambiguously identify the fluorinated BPA demonstrating the ability of the protocol to be not only rapid (30 seconds) at tagging the BPA, but successful at detecting this endocrine disrupting compound at such low concentration (Fig. 5). The method detection limit (MDL) for the *bis*-difluoromethylated BPA in this soil was determined to be 0.01 μg.mL^−1^ (Supporting Information, Page S4).

## Methods

### Materials

All chemicals were purchased from commercial suppliers and used as received. Acetonitrile, methylene chloride, Bisphenol E, Bis-(4-hydroxyphenyl)methane (Bisphenol F), Bisphenol BP, Bisphenol G, 4,4′-(9-fluorenylidene)diphenol (Bisphenol FL), 3-pentanol, 2-methyl-2-pentanol, 2,2′-thiodiethanol (thiodiglycol), pinacolyl alcohol (3,3-dimethyl-2-butanol), 3,7-dimethyl-3-octanol, and 2-dimethylaminoethanol were purchased from Sigma-Aldrich chemicals (St. Louis, MO). Bisphenol A was purchased from Spectrum Laboratory Products (Gardena, CA). Bis-(4-hydroxyphenyl) sulfone (Bisphenol S), 1,1-Bis-(4-hydroxyphenyl)cyclohexane (Bisphenol Z, >98%), 4,4′-(1-phenylethylidine)bisphenol (Bisphenol AP), 2,2-Bis-(4-hydroxy-3-methylphenyl)propane (Bisphenol C, >98%), and 2,2-Bis-(4-hydroxyphenyl)hexafluoropropane (Bisphenol AF) were purchased from TCI America (Portland, OR). Diethyl (bromodifluoromethyl) phosphonate was purchased from Matrix Scientific Inc. (Columbia, SC). Deuterated acetonitrile (CD_3_CN) was purchased from Alfa Aesar (Ward Hill, MA). Acrodisc PTFE syringe filters (0.45 μm) were purchased from Pall laboratories (Port Washington, NY.). Nebraska (EPA standard 54–135–4) soil was obtained from the soil matrix library at the Forensic Science Center in the Lawrence Livermore National Laboratory. All new *bis*-difluoromethylated bisphenols were purified using a Biotage Isolera flash column chromatography purification system using SNAP KP-Si gel column cartridges. HRMS analyses were obtained in the Forensic Science Center at the Lawrence Livermore National Laboratory using chemical ionization (CI). Combustion analyses were conducted at Galbraith Laboratories (Knoxville, TN).

### EI-GC-MS experiments

EI-GC-MS analyses were carried out employing a 6890 Agilent GC equipped with a 5975 MS detector featuring a split/splitless injector^23,33^. The column used for our analyses was an Agilent DB-5MS capillary column (dimensions: 30 m × 0.25 mm i.d. × 0.25 µm i.f.). Ultra-high purity helium was employed as the carrier gas at a flow rate of 0.8 mL.min^−1^. The injector temperature was set to 250 °C, and the injection volume was 1 µL. The oven temperature program used for the work was the following: 40 °C (held for t = 3 min), increased at a rate of 8 °C.min^−1^ to 300 °C and then held for t = 3 min. The MS ion source and quadrupole temperatures were set at 230 °C and 150 °C, respectively. The electron ionization energy used was 70 eV. The MS was operated to scan from *m/z* = 29 to *m/z* = 600 in t = 0.4 sec.

### Derivatization protocol of individual bisphenols

The initial protocol conditions included longer reaction times and stirring rather than vortexing aimed at the definitive derivatization of the panel of 11 BPs. Thus, the bisphenol (0.04 mmol) was placed in a glass autosampler vial equipped with a small stir bar and treated sequentially via pipette with 0.1 M KOH/H_2_O (800 μL), acetonitrile (CH_3_CN, 800 μL) and diethyl (bromodifluoromethyl) phosphonate (DBDFP, 21.4 μL, 0.12 mmol, 3.0 equiv. to bisphenol). The vial was capped and stirred vigorously at ambient temperature for 5 minutes. After the stirring was finalized, the mixture was allowed to stand to reveal a biphasic mixture and 500 μL of the clear, top layer (acetonitrile) was aliquoted into another glass autosampler vial containing anhydrous sodium sulfate (50 mg). The dried, organic fraction was passed through a syringe PTFE filter disc (0.45 μm) and 20 μL of the filtrate were aliquoted and diluted to 1.5 mL total volume with methylene chloride in an autosampler vial for GC-MS analysis.

### Assessing the selectivity of the derivatization of bisphenol A in a mixture of aliphatic alcohols

In a 2 mL glass scintillation vial equipped with a stir bar, a 500 μL stock solution of seven alcohols, six of them aliphatic (3-pentanol, 2-methyl-2-pentanol, 2,2-thiodiethanol, pinacolyl alcohol, 3,7-dimethyl-3-octanol and *N,N*-dimethylaminoethanol) and BPA (each one at a 1000 μg mL^−1^ concentration) were treated sequentially with 0.1 M KOH/H_2_O (800 μL), acetonitrile (800 μL) and DBDFP (50 μL). The resulting biphasic mixture was vigorously stirred for 5 minutes at ambient temperature. After the stirring was stopped and the biphasic mixture revealed, 20 μL of the top layer (acetonitrile) was aliquoted into another autosampler vial and diluted to 1.5 mL with methylene chloride for GC-MS analysis. The extended period of time (5 min.) in contrast to the established protocol (Sections 2.4. and on) was to ensure that the reaction was very selective and non-reactive over the other alcohols.

### Detection of bisphenol A in Nebraska soil sample at a 10 μg.g^−1^ concentration

Three sets of Nebraska soil samples (100 mg) in 4 mL vials were spiked with a BPA solution (1 μg mL^−1^) in methylene chloride and mixed, via tumbling, using a rotary evaporator at 40 °C for 15 minutes that after fully drying leads to a 10 μg g^−1^ BPA-contaminated soil. The contaminated soil was treated with a 0.1 M KOH aqueous solution (800 μL), followed by the sequential addition of acetonitrile (800 μL) and DBDFP (30 μL). The vials were capped, and the resulting biphasic suspensions were each mixed using a vortex for 30 seconds. After this time, 800 μL of the organic, top layer was aliquoted into an autosampler vial and dried with anhydrous sodium sulfate (30 mg). After drying, 100 μL of the organic phase was aliquoted into an autosampler vial equipped with a glass insert for GC-MS analysis.

### Direct derivatization protocol for bisphenol A-treated Nebraska soil spiked at 1 μg.g^−1^ concentration

Nebraska soil (100 mg) was placed in a 4 mL vial and spiked with a BPA solution (0.1 μg mL^−1^) in methylene chloride. The suspension was mixed, via tumbling, using a rotary evaporator at 40 °C for 15 minutes that after fully drying leads to a 1 μg g^−1^ BPA-contaminated soil sample. The soil was treated using a pipette with 0.1 M KOH/H_2_O (1 mL), and then sequentially with acetonitrile (1 mL) and DBDFP (30 μL). The mixture was vortexed for 30 seconds at ambient temperature after which time an aliquot was extracted via pipette (800 μL), dried over anhydrous sodium sulfate (Na_2_SO_4_, 30 mg) and transferred (100 μL) to an autosampler vial equipped with a glass insert for GC-MS analysis. This set of experiments were conducted in triplicates.

### General procedure for the synthesis of *bis*-difluoromethylated bisphenols

The bisphenol (0.84 mmol) is dissolved in CH_3_CN (3 mL) and then treated sequentially with DBDFP (500 μL, 2.8 mmol, 3.3 equiv. to bisphenol) followed by 0.1 M KOH/H_2_O (pH 12.8, 3 mL). The resulting mixture was stirred at ambient temperature for 30 minutes. The acetonitrile layer was dried over Na_2_SO_4_, evaporated *in vacuo* and purified by flash column chromatography (hexanes → 7:3 EtOAc/hexanes) to furnish the *bis*-difluoromethyl-bisphenol. All characterization data associated for each *bis*-difluoromethyl-bisphenol used in this study can be found in the SI section of this manuscript.

## Conclusions

The methodology described herein represents the first expedient and practical derivatization of BPs via difluoromethylation for their enhanced and unequivocal detection by GC-MS. A remarkable aspect of the protocol is the fact that for the determination of BPs in soil, there are no prior extraction/concentration steps involved, thus greatly speeding up the process by removing the sample preparation step from the analysis. The fast and direct derivatization of BPA in Nebraska soil (at 1 μg.g^−1^) showcases the protocol’s practicality and establishes it as a strong derivatization tool in the analytical chemist’s toolbox for these types of compounds.

## Supplementary information

Supplementary Information
