# Supplementary material for: Carbene-based Difluoromethylation of Bisphenols: Application to the Instantaneous Tagging of Bisphenol A in Spiked Soil for Its Detection and Identification by Electron Ionization Gas Chromatography-Mass Spectrometry
Source: Sci Rep. 2019 Nov 22;9:17360. doi: 10.1038/s41598-019-53735-9 (PMC6874605; doi:10.1038/s41598-019-53735-9)
Supplement: Supplementary file 1 — Supplementary Information [file 41598_2019_53735_MOESM1_ESM.docx]

**-Supporting Information-**

“Carbene-based Difluoromethylation of Bisphenols: Application to the Instantaneous Tagging of Bisphenol A in Spiked Soils for Its Detection and Identification by Gas Chromatography-Mass Spectrometry”

**-Table of Contents-**

**Page**

General protocols for derivatizations of bisphenols S2

NMR Stability Studies on Bisphenol-A *bis*-difluoromethylated analog S2

Figure 1. NMR stability study spectra on BPA-(CF_2_H)_2_ S3

General procedure for the difluoromethylation reaction of isolated bisphenols S4

General procedure for the difluoromethylation reaction of BPA in soils (10 μg^.^g^-1^) S4

MDL calibration curve determination for bisphenol-A (CF_2_H)_2_ S4

Figure 2. General difluoromethylation scheme for all bisphenols used in this work S5

Figure 3. GC-MS of Bisphenol A S6

Figure 4. GC-MS of Bisphenol A (CF_2_H)_2_ S7

Figure 5. GC-MS of Bisphenol S S8

Figure 6. GC-MS of Bisphenol S (CF_2_H)_2_ S9

Figure 7. GC-MS of Bisphenol G S10

Figure 8. GC-MS of Bisphenol G (CF_2_H)_2_ S11

Figure 9. GC-MS of Bisphenol Z S12

Figure 10. GC-MS of Bisphenol Z (CF_2_H)_2_ S13

Figure 11. GC-MS of Bisphenol C S14

Figure 12. GC-MS of Bisphenol C (CF_2_H)_2_ S15

Figure 13. GC-MS of Bisphenol AF S16

Figure 14. GC-MS of Bisphenol AF (CF_2_H)_2_ S17

Figure 15. GC-MS of Bisphenol FL S18

Figure 16. GC-MS of Bisphenol FL (CF_2_H)_2_ S19

Figure 17. GC-MS of Bisphenol AP S20

Figure 18. GC-MS of Bisphenol AP (CF_2_H)_2_ S21

Figure 19. GC-MS of Bisphenol BP S22

Figure 20. GC-MS of Bisphenol BP (CF_2_H)_2_ S23

Figure 21. GC-MS of Bisphenol E S24

Figure 22. GC-MS of Bisphenol E (CF_2_H)_2_ S25

Figure 23. GC-MS of Bisphenol F S26

Figure 24. GC-MS of Bisphenol F (CF_2_H)_2_ S27

Characterization of newly derivatized bisphenols S28-S31

NMR spectra for all derivatized bisphenols (Figures 25-35) S32-S43

**General procedure for the synthesis of *bis*-difluoromethylated bisphenols**

In a 20 mL glass scintillation vial equipped with a stir bar, the bisphenol (0.84 mmol) was taken up in CH_3_CN (3 mL) and treated sequentially with DBDFP (500 μL, 2.8 mmol, 3.3 equiv. to bisphenol) and 5 M KOH/H_2_O (3 mL). The resulting mixture was stirred at ambient temperature for 10 minutes. The acetonitrile layer was dried over Na_2_SO_4_, evaporated *in vacuo* in a rotary evaporator and purified by flash column chromatography (ethyl acetate 0 🡪 80% ethyl acetate/hexanes) to furnish the *bis*-difluoromethyl bisphenol as a white/off white solid.

**NMR Stability Studies on Bisphenol-A *bis*-difluoromethylated analog**

The NMR stability study of the final, derivatized product of bisphenol A by the protocol described in this work was conducted in CDCl_3_. The study was aimed to evaluate the overall stability of the products generated by the protocol if kept in a similar solvent (*e.g.* dichloromethane) over extended periods of time. As alluded to in the paper, the stability of the O-CF_2_H should be superior to that exhibited by the O-CH_3_ group based solely on inductive effects from the fluorine atoms which would discourage the formation of a carbocationic species generated from the hydrolysis of this group. Furthermore, there will be reduced nucleophilic attack on the carbon in the CF_2_H moiety relative to that of the O-CH_3_ moiety as a result of the electronegativity of the fluorine atoms. Thus, 20 mgs of the material was dissolved in 500 μL of CDCl_3_ and placed in a NMR tube. ^1^H NMR acquisitions were obtained at t = 0.1, 8, 24, 48, 72, 96, and 120 hours. All spectra were recorded at 30.0 ± 0.1 ^o^C.

**Figure 1.** ^1^H NMR showing the stability of the BPA-(CF_2_H)_2_ derivative in CDCl_3_ over a period of five days at 30 ^o^C. A) 1H NMR of BPA(CF2H)2 showing the enlarged area that was followed during the stability study. The triplet centered at 6.5 ppm corresponds to the proton in the difluoromethyl moiety, B) No hydrolysis or degradation of the material is observed over 5 days, showing the expected high stability of the derivatized bisphenols described in this work.

**General procedure for the difluoromethylation reaction of isolated bisphenols**

The bisphenol (0.04 mmol) was placed in a glass autosampler vial equipped with a small stir bar and treated sequentially via pipette with 5 M KOH/H_2_O (800 μL), acetonitrile (CH_3_CN, 800 μL) and diethyl (bromodifluoromethyl) phosphonate (DBDFP, 21.4 μL, 0.12 mmol, 3.0 equiv. to bisphenol). The vial was capped and stirred vigorously at ambient temperature for 5 minutes. After the stirring was finalized, the mixture was allowed to stand to reveal a biphasic mixture and 500 mL of the clear, top layer (acetonitrile) was aliquoted into another glass autosampler vial containing anhydrous sodium sulfate (50 mg). The dried, organic fraction was passed through a syringe PTFE filter disc (0.45 μm) and 20 μL of the filtrate were aliquoted and diluted to 1.5 mL total volume with methylene chloride in an autosampler vial for GC-MS analysis.

**General procedure for the difluoromethylation reaction of bisphenol A in soils (10 μg^.^g^-1^)**

Three sets of the chosen soil sample (100 mg) in 4 mL vials were spiked with a BPA solution (1 μg^.^mL^-1^) in methylene chloride and mixed, via tumbling, using a rotary evaporator at 40 ^o^C for 15 minutes that after fully drying leads to a 10 μg g^-1^ BPA-contaminated soil. The contaminated soil was treated with a 5M KOH aqueous solution (800 μL), followed by the sequential addition of acetonitrile (800 μL) and DBDFP (30 μL). The vials were closed and the resulting biphasic suspensions each mixed using a vortex for 30 seconds. After this time, 800 μL of the organic, top layer was aliquoted into an autosampler vial and dried with anhydrous sodium sulfate (30 mg). After drying, 100 μL of the organic phase was transferred into an autosampler vial equipped with a glass insert for GC-MS analysis.

**MDL calibration curve for *bis*-difluoromethylated bisphenol A (Nebraska soil)**

The method detection limit (MDL) for BPA(CF_2_H)_2_ was determined to be 0.01 μg/mL (7 replicates using the single-tailed 99^th^ percentile *t* statistic). The MDL was determined following the guidelines in EPA document EPA 821-R-16-006 “Definition and Procedure for the Determination of the Method Detection Limit, Revision 2,” December, 2016. A linear 8-point calibration curve was generated for this study, covering a BPA(CF_2_H)_2_ calibration range from 0.1 to 6 ug/mL (forced line through 0,0; y = 1.23E-6(x); R^2^=0.999.

**Figure 2.** Chemical structures of all bisphenols and their bis(difluoromethylated) counterparts described in this paper. Bisphenol A (BPA) and its fluorinated analog BPA(CF_2_H)_2_, used in the soil experiments are highlighted with a box.


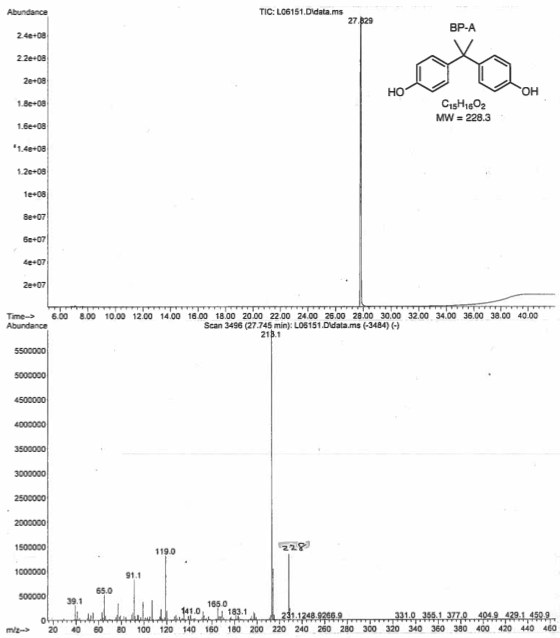


**Figure 3.** GC-MS of Bisphenol A.


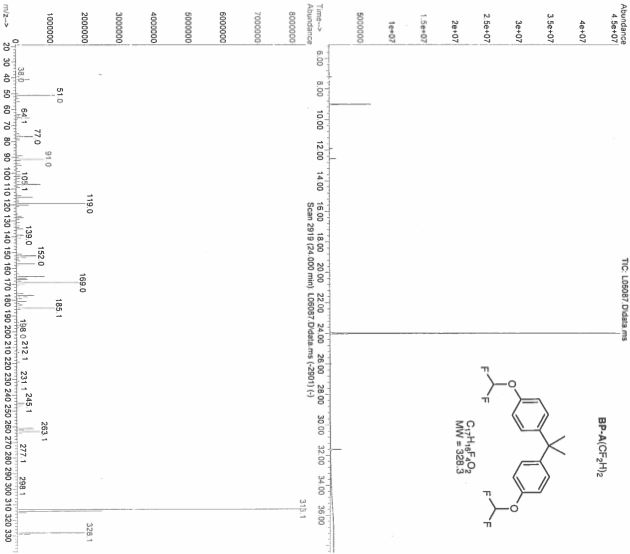


**Figure 4.** GC-MS of Bisphenol A (CF_2_H)_2_.


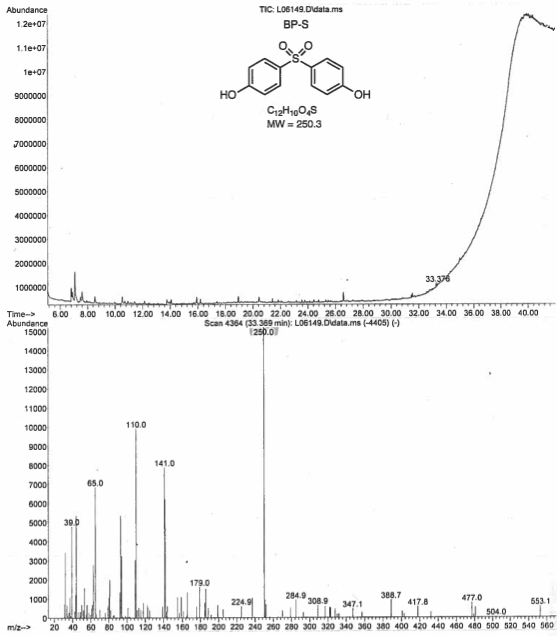


**Figure 5.** GC-MS of Bisphenol S.


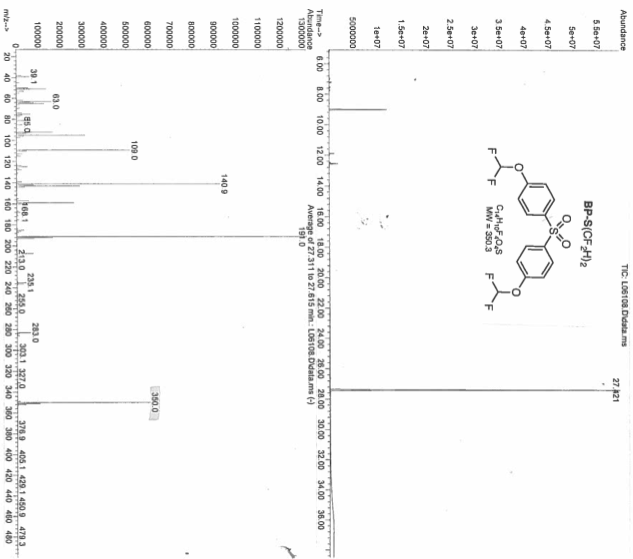


**Figure 6.** GC-MS of Bisphenol S (CF_2_H)_2_.


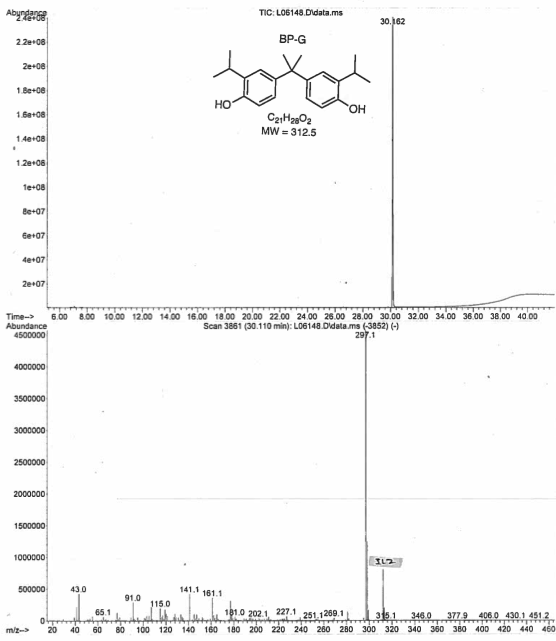


**Figure 7.** GC-MS of Bisphenol G.


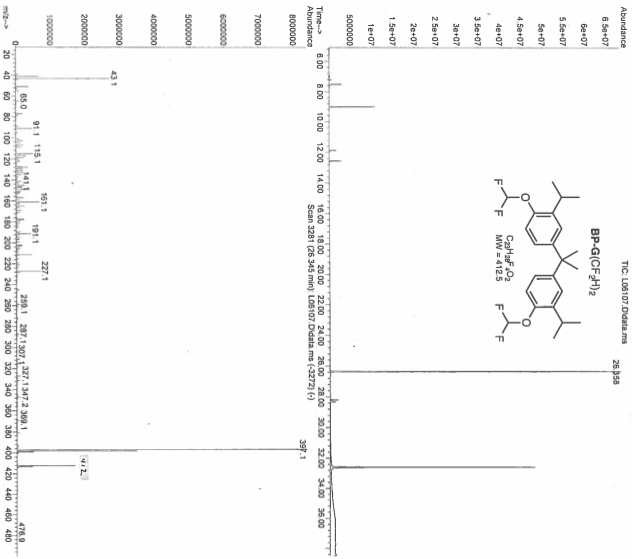


**Figure 8.** GC-MS of Bisphenol G (CF_2_H)_2_.


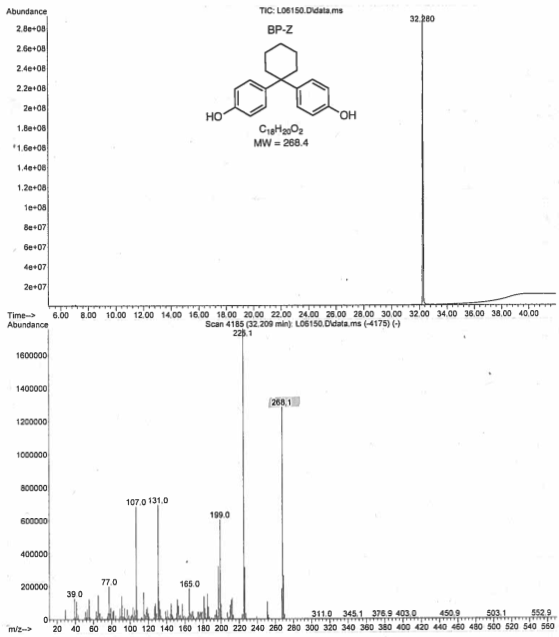


**Figure 9.** GC-MS of Bisphenol Z.


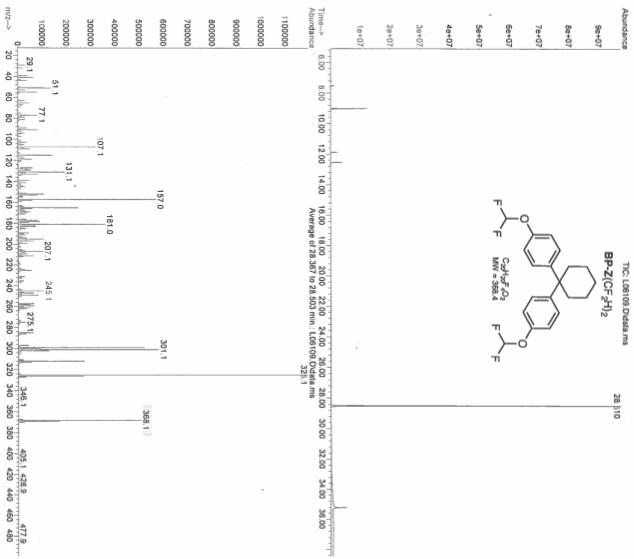


**Figure 10.** GC-MS of Bisphenol Z (CF_2_H)_2_.


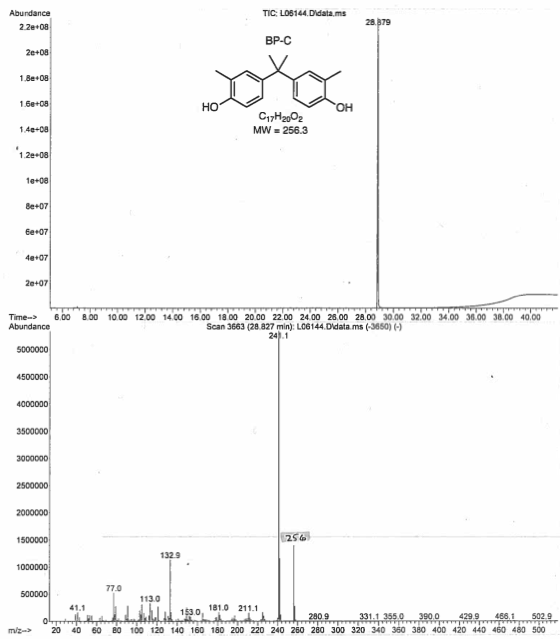


**Figure 11.** GC-MS of Bisphenol C.


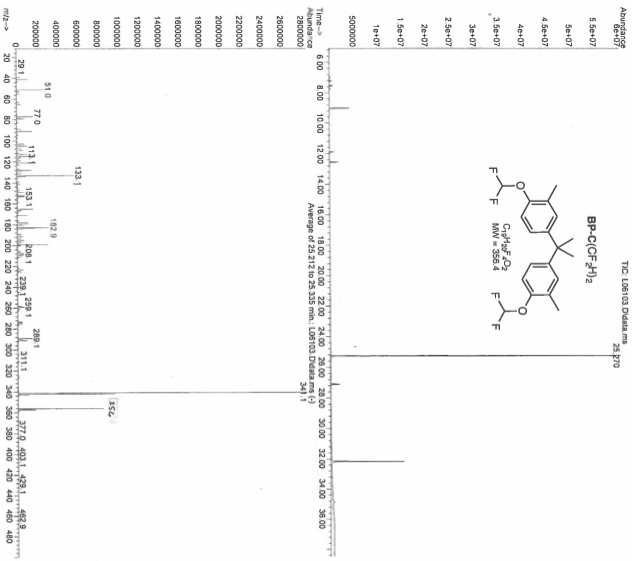


**Figure 12.** GC-MS of Bisphenol C (CF_2_H)_2_.


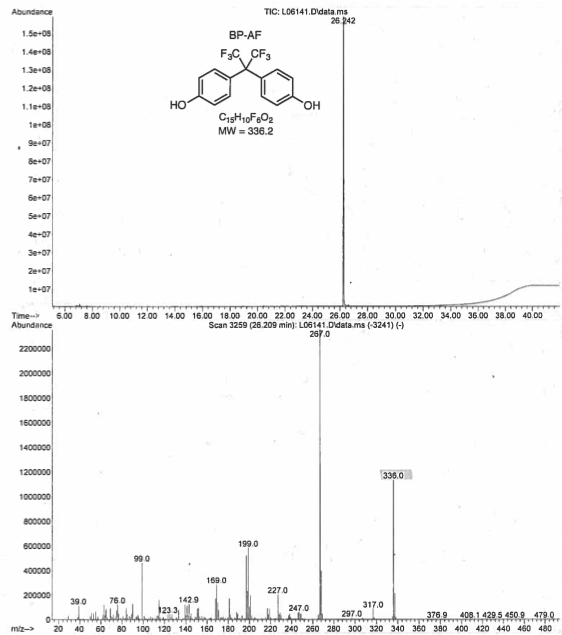


**Figure 13.** GC-MS of Bisphenol AF.


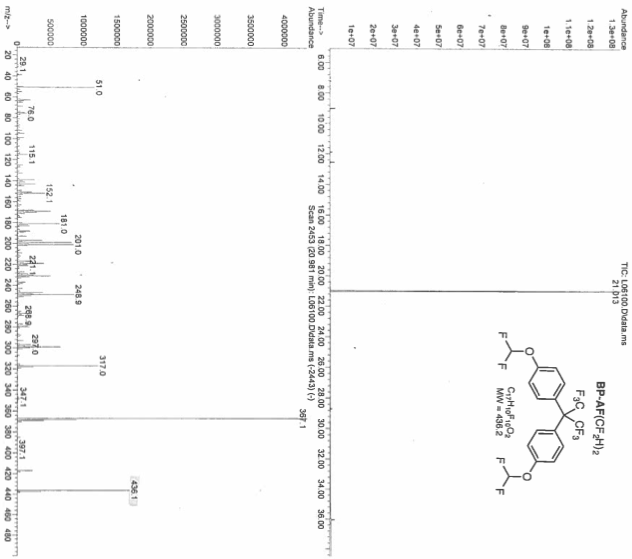


**Figure 14.** GC-MS of Bisphenol AF (CF_2_H)_2_.


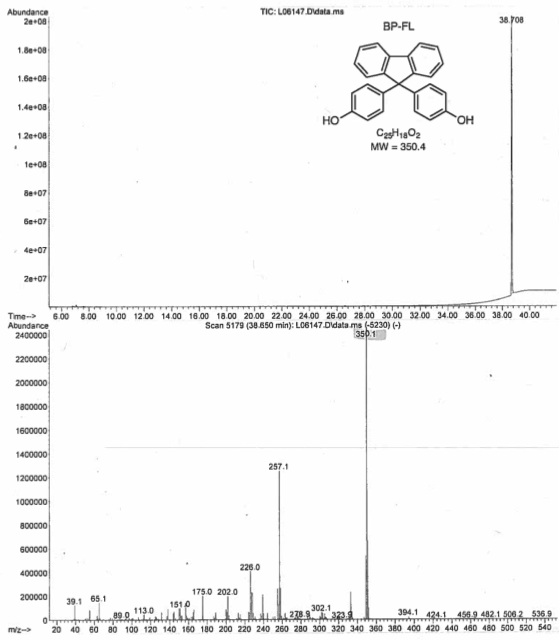


**Figure 15.** GC-MS of Bisphenol FL.


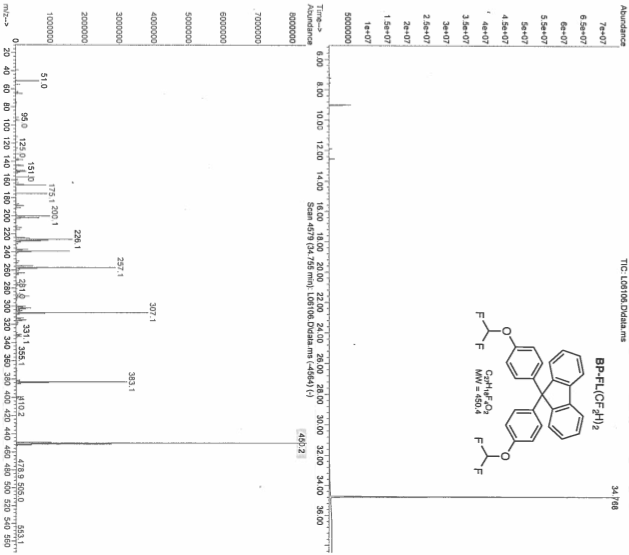


**Figure 16.** GC-MS of Bisphenol F (CF_2_H)_2_.


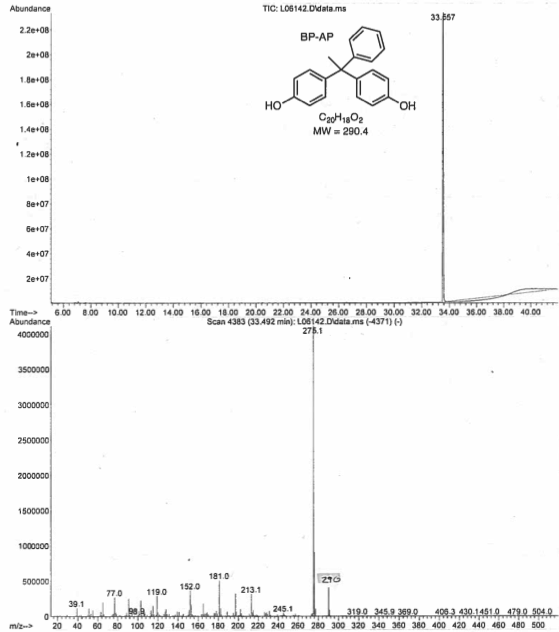


**Figure 17.** GC-MS of Bisphenol AP.


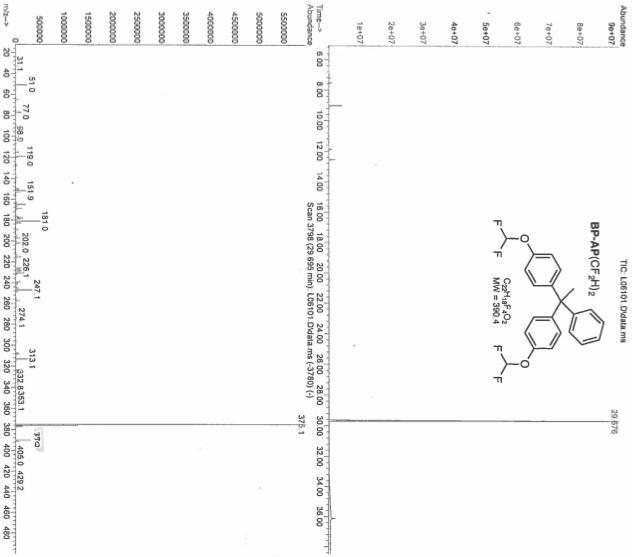


**Figure 18.** GC-MS of Bisphenol AP (CF_2_H)_2_.


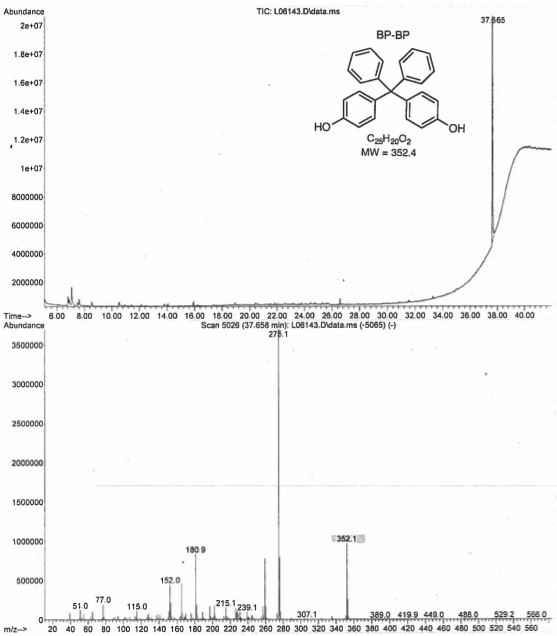


**Figure 19.** GC-MS of Bisphenol BP.


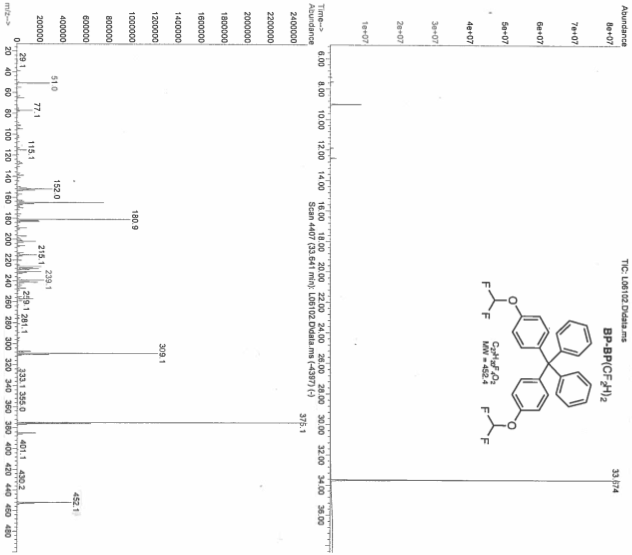


**Figure 20.** GC-MS of Bisphenol BP (CF_2_H)_2_.


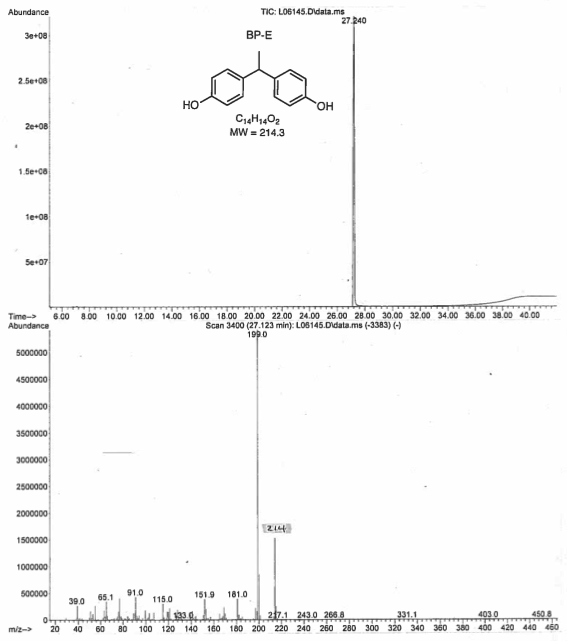


**Figure 21.** GC-MS of Bisphenol E.

**
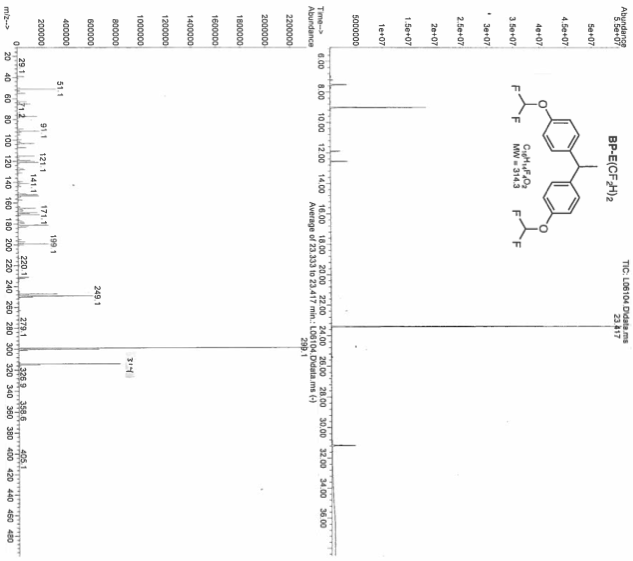
**

**Figure 22.** GC-MS of Bisphenol E (CF_2_H)_2_.


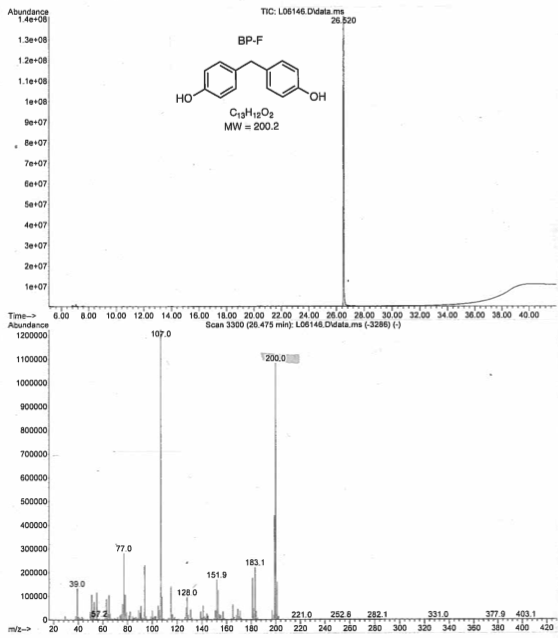


**Figure 23.** GC-MS of Bisphenol F.


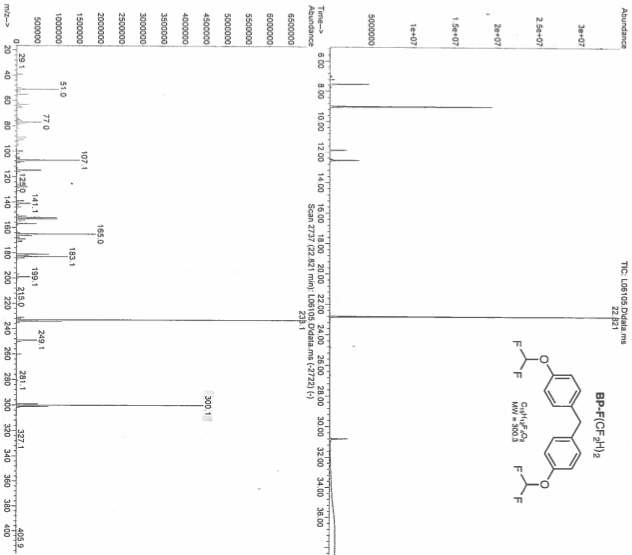


**Figure 24.** GC-MS of Bisphenol F (CF_2_H)_2_.

**Characterization of newly derivatized bisphenols**

**Nuclear Magnetic Resonance**

^1^H NMR (600 MHz), ^13^C NMR (150 MHz) and ^19^F NMR (565 MHz) were all recorded in CDCl_3_. Spectra were obtained using a Bruker Avance III 600 MHz instrument equipped with a Bruker TCI 5 mm cryoprobe (Bruker Biospin, Billerica, MA) at 30.0 ± 0.1 ^o^C. NMR data is reported as follows: chemical shift (δ) (parts per million, ppm); multiplicity: s (singlet), d (doublet), t (triplet), q (quartet), sep (septet), dd (doublet of doublets) and br (broad) and m (multiplet, given as a ppm range); coupling constants (*J*) are given in Hertz (Hz). ^1^H NMR chemical shifts are calibrated with respect to residual CDCl_3_ (δ = 7.26 ppm), whereas for ^13^C NMR, the center peak for CDCl_3_ (δ = 77.0 ppm), were used for the calibration.

**BP-A(CF_2_H)_2_:** Obtained as a white solid (Yield: 77%); R_f_ = 0.56 (1:1 EtOAc/hexanes); ^1^H NMR (CDCl_3_, 600 MHz) δ 7.23 (d, *J* = 8.4 Hz, 4H), 7.04 (d, *J* = 8.4 Hz, 4H), 6.50 (t, *J*_H-F_ = 254.5 Hz, 2H, 2 x OC*H*F_2_), 1.67 (s, 6H, 2 x *Me*); ^13^C NMR (151 MHz) δ 149.2, 147.6, 128.2, 118.6, 116.9 (t, *J*_C-F_ = 256.7 Hz), 41.9, 30.9; ^19^F NMR (565 MHz) δ -83.1; HRMS (CI) *m/z* calcd. for C_17_H_16_F_4_O_2_ [M^+^**^.^**]: 328.3066; found 328.3045; Anal. Calcd for C_17_H_16_F_4_O_2_: C, 62.19; H, 4.91. Found: C, 62.08; H, 4.94.

**BP-S(CF_2_H)_2_:** Obtained as an off-white solid (Yield: 85%); R_f_ = 0.52 (1:1 EtOAc/hexanes); ^1^H NMR (CDCl_3_, 600 MHz) δ 7.93 (d, *J* = 8.0 Hz, 4H), 7.22 (d, *J* = 8.0 Hz, 4H), 6.52 (t, *J*_H-F_ = 255.0 Hz, 2H, 2 x OC*H*F_2_); ^13^C NMR (151 MHz) δ 154.2, 137.4, 130.5, 119.6, 115.3 (t, *J*_C-F_ = 255.8 Hz); ^19^F NMR (565 MHz) δ -84.0; HRMS (CI) *m/z* calcd. for C_14_H_10_F_4_O_4_S [M^+^**^.^**]: 350.0236; found 350.0233. Anal. Calcd for C_15_H_12_F_4_O_2_: C, 48.01; H, 2.88. Found: C, 47.94; H, 2.87.

**BP-G(CF_2_H)_2_:** Obtained as a white solid (Yield: 68%); R_f_ = 0.55 (1:4 EtOAc/hexanes); ^1^H NMR (CDCl_3_, 600 MHz) δ 7.06 (s, 2H), 6.94 (d, *J* = 8.4 Hz, 2H), 6.67 (d, *J* = 8.4 Hz, 2H), 6.50 (t, *J*_H-F_ = 254.5 Hz, 2H, 2 x OC*H*F_2_), 3.19 (sep, *J* = 7.0 Hz, 2H), 1.62 (s, 6H, 2 x *Me*), 1.19 (d, *J* = 7.0 Hz, 12H); ^13^C NMR (151 MHz) δ 151.4, 143.3, 133.6, 126.3, 115.4, 114.8 (t, *J*_C-F_ = 256.7 Hz), 42.6, 30.8, 26.9, 21.9; ^19^F NMR (565 MHz) δ -83.1; HRMS (CI) *m/z* calcd. for C_23_H_28_F_4_O_2_ [M^+^**^.^**]: 412.2025; found 412.2019. Anal. Calcd for C_23_H_28_F_4_O_2_: C, 66.98; H, 6.84. Found: C, 66.92; H, 6.77.

**BP-Z(CF_2_H)_2_:** Obtained as a white, flaky solid (Yield: 84%); R_f_ = 0.81 (1:1 EtOAc/hexanes); ^1^H NMR (CDCl_3_, 600 MHz) δ 7.23 (d, *J* = 8.4 Hz, 4H), 6.77 (d, *J* = 8.4 Hz, 4H), 6.47 (t, *J*_H-F_ = 254.4 Hz, 2H, 2 x OC*H*F_2_), 2.10-2.18 (m, 4H), 1.53-1.42 (m, 6H); ^13^C NMR (151 MHz) δ 154.2, 142.6, 128.3, 114.8 (t, *J*_C-F_ = 256.6 Hz), 114.6, 45.5, 38.0, 26.4, 22.1; ^19^F NMR (565 MHz) δ -82.8; HRMS (CI) *m/z* calcd. for C_20_H_20_F_4_O_2_ [M^+^**^.^**]: 368.1399; found 368.1392. Anal. Calcd for C_20_H_20_F_4_O_2_: C, 65.21; H, 5.47. Found: C, 65.19; H, 5.34.

**BP-C(CF_2_H)_2_:** Obtained as an off-white solid (Yield: 83%); R_f_ = 0.64 (1:1 EtOAc/hexanes); ^1^H NMR (CDCl_3_, 600 MHz) δ 6.97 (s, 2H), 6.89 (d, *J* = 8.4 Hz, 2H), 6.66 (d, *J* = 8.4 Hz, 2H), 6.53 (t, *J*_H-F_ = 254.5 Hz, 2H, 2 x OC*H*F_2_), 2.41 (s, 6H), 1.62 (s, 6H); ^13^C NMR (151 MHz) δ 151.8, 144.3, 129.0, 126.3,123.5, 117.3 (t, *J*_C-F_ = 256.7 Hz), 117.0, 40.9, 30.8, 15.8; ^19^F NMR (565 MHz) δ -82.8; HRMS (CI) *m/z* calcd. for C_19_H_20_F_4_O_2_ [M^+^**^.^**]: 356.1399; found 356.1396. Anal. Calcd for C_19_H_20_F_4_O_2_: C, 64.04; H, 5.66. Found: C, 63.90; H, 5.48.

**BP-AF(CF_2_H)_2_:** Obtained as a white, flaky solid (Yield: 78%); R_f_ = 0.70 (1:1 EtOAc/hexanes); ^1^H NMR (CDCl_3_, 600 MHz) δ 7.31 (d, *J* = 8.3 Hz, 4H), 6.83 (d, *J* = 8.3 Hz, 4H), 6.52 (t, *J*_H-F_ = 254.3 Hz, 2H, 2 x OC*H*F_2_); ^13^C NMR (151 MHz) δ 156.4, 131.6, 126.4, 125.1 (q, *J* = 285.4 Hz, 2 x *CF_3_*), 117.3 (t, *J*_C-F_ = 256.7 Hz), 115.2, 63.4; ^19^F NMR (565 MHz) δ -82.8 (2 x OCF_2_H), -64.8 (2 x CF_3_); HRMS (CI) *m/z* calcd. for C_17_H_10_F_10_O_2_ [M^+^**^.^**]: 436.0521; found 436.0519; Anal. Calcd for C_17_H_10_F_10_O_2_: C, 46.81; H, 2.31. Found: C, 46.68; H, 2.15.

**BP-FL(CF_2_H)_2_:** Obtained as an off-white solid (Yield: 78%); R_f_ = 0.47 (1:4 EtOAc/hexanes); ^1^H NMR (CDCl_3_, 600 MHz) δ 7.83 (d, *J* = 7.5 Hz, 2H), 7.41 (m, 2H), 7.32 (t, *J* = 7.5 Hz, 2H), 7.01 (d, *J* = 8.1 Hz, 4H), 6.57 (d, *J* = 8.1 Hz, 4H), 6.49 (t, *J*_H-F_ = 254.2 Hz, 2H, 2 x OC*H*F_2_); ^13^C NMR (151 MHz) δ 155.0, 151.9, 139.9, 137.5, 129.3, 127.7, 127.2, 126.2, 114.5 (t, *J*_C-F_ = 256.7 Hz), 114.3, 64.1; ^19^F NMR (565 MHz) δ -83.9; HRMS (CI) *m/z* calcd. for C_27_H_18_F_4_O_2_ [M^+^**^.^**]: 450.1243; found 450.1241. Anal. Calcd for C_15_H_12_F_4_O_2_: C, 72.00; H, 4.03. Found: C, 71.88; H, 3.96.

**BP-AP(CF_2_H)_2_:** Obtained as a white solid (Yield: 71%); R_f_ = 0.80 (1:1 EtOAc/hexanes); ^1^H NMR (CDCl_3_, 600 MHz) δ 7.26-7.20 (m, 3H), 7.11-7.07 (m, 2H), 6.95-6.92 (m, 4H), 6.79-6.73 (m, 4H), 6.44 (t, *J*_H-F_ = 254.6 Hz, 2H, 2 x OC*H*F_2_), 2.09 (s, 3H); ^13^C NMR (151 MHz) δ 154.2, 148.8, 141.4, 130.1, 128.8, 128.4, 126.2, 118.0 (t, *J*_C-F_ = 256.4 Hz), 114.2, 50.9, 30.8; ^19^F NMR (565 MHz) δ -83.3; HRMS (CI) *m/z* calcd. for C_22_H_18_F_4_O_2_ [M^+^**^.^**]: 390.1243; found 390.1239. Anal. Calcd for C_22_H_18_F_4_O_2_: C, 67.69; H, 4.65. Found: C, 67.57; H, 4.44.

**BP-BP(CF_2_H)_2_:** Obtained as a white, solid (Yield: 79%); R_f_ = 0.75 (1:4 EtOAc/hexanes); ^1^H NMR (CDCl_3_, 600 MHz) δ 7.26-7.17 (m, 10H), 7.09 (d, *J* = 8.3 Hz, 4H), 6.70 (d, *J* = 8.3 Hz, 4H), 6.55 (t, *J*_H-F_ = 254.4 Hz, 2H, 2 x OC*H*F_2_); ^13^C NMR (151 MHz) δ 153.8, 147.7, 139.1, 132.0, 131.1, 131.0, 128.4, 128.3, 126.2, 126.0, 117.5 (t, *J*_C-F_ = 256.6 Hz), 115.2, 43.3; ^19^F NMR (565 MHz) δ -83.2; HRMS (CI) *m/z* calcd. for C_27_H_20_F_4_O_2_ [M^+^**^.^**]: 452.1399; found 452.1398. Anal. Calcd for C_27_H_20_F_4_O_2_: C, 71.68; H, 4.46. Found: C, 71.61; H, 4.42.

**BP-E(CF_2_H)_2_:** Obtained as a white solid (Yield: 66%); R_f_ = 0.54 (1:1 EtOAc/hexanes); ^1^H NMR (CDCl_3_, 600 MHz) δ 7.07 (d, *J* = 8.1 Hz, 4H), 6.80 (d, *J* = 8.1 Hz, 4H), 6.57 (t, *J*_H-F_ = 254.4 Hz, 2H, 2 x OC*H*F_2_), 4.03 (q, *J* = 7.2 Hz, 1H), 1.53 (d, *J* = 7.2 Hz, 3H); ^13^C NMR (151 MHz) δ 150.1 (t, *J*_C-F_ = 2.4 Hz), 147.5, 128.1, 118.6, 116.4 (t, *J*_C-F_ = 256.6 Hz), 42.8, 16.3; ^19^F NMR (565 MHz) δ -83.0; HRMS (CI) *m/z* calcd. for C_16_H_14_F_4_O_2_ [M^+^**^.^**]: 314.0930; found 314.0921. Anal. Calcd for C_16_H_14_F_4_O_2_: C, 61.15; H, 4.49. Found: C, 61.02; H, 4.28.

**BP-F(CF_2_H)_2_**: Obtained as a white, flaky solid (Yield: 80%); R_f_ = 0.55 (1:1 EtOAc/hexanes); ^1^H NMR (CDCl_3_, 600 MHz) δ 7.06 (d, *J* = 8.5 Hz, 4H), 6.78 (d, *J* = 8.5 Hz, 4H), 6.50 (t, *J*_H-F_ = 254.6 Hz, 2H, 2 x OC*H*F_2_), 3.83 (s, 2H); ^13^C NMR (151 MHz) δ 154.1, 134.5, 130.0, 115.4, 115.0 (t, *J*_C-F_ = 256.5 Hz), 40.2; ^19^F NMR (565 MHz) δ -83.0; HRMS (CI) *m/z* calcd. for C_15_H_12_F_4_O_2_ [M^+^**^.^**]: 300.0773; found 300.0768. Anal. Calcd for C_15_H_12_F_4_O_2_: C, 60.00; H, 4.03. Found: C, 59.94; H, 3.89.


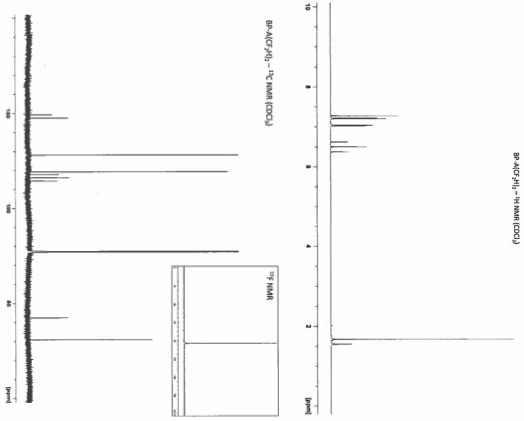


**Figure 25.** NMR spectra for Bisphenol A (CF_2_H)_2_.


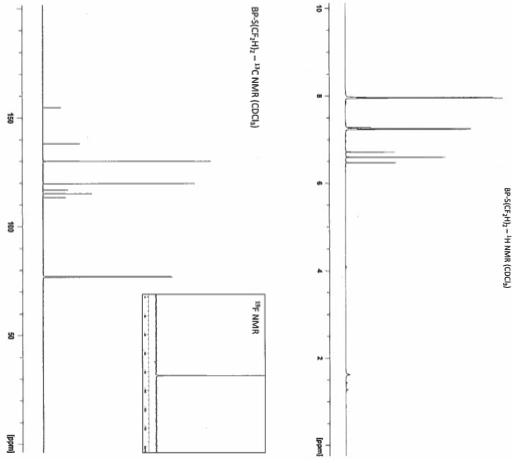


**Figure 26.** NMR spectra for Bisphenol S (CF_2_H)_2_.


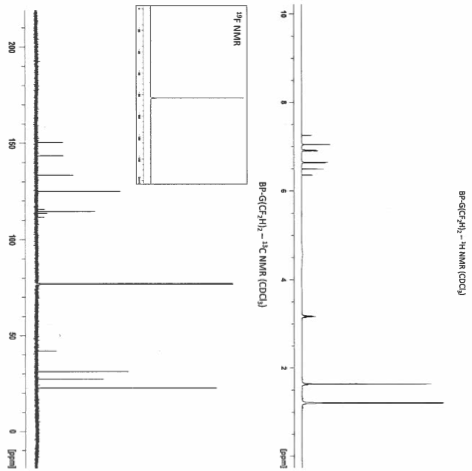


**Figure 27.** NMR spectra for Bisphenol G (CF_2_H)_2_.


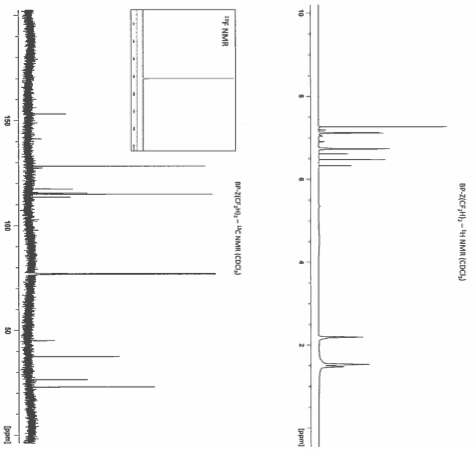


**Figure 28.** NMR spectra for Bisphenol Z (CF_2_H)_2_.


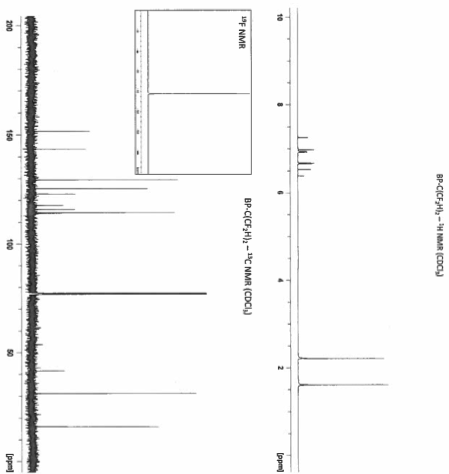


**Figure 29.** NMR spectra for Bisphenol C (CF_2_H)_2_.


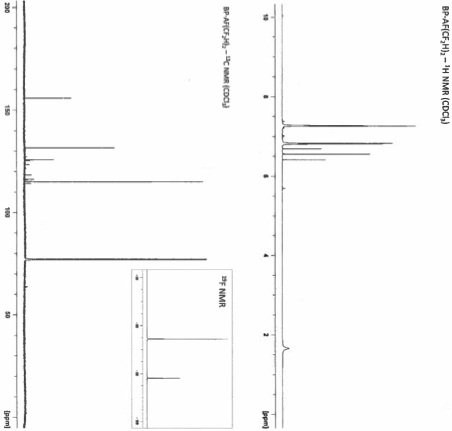


**Figure 30.** NMR spectra for Bisphenol AF (CF_2_H)_2_.


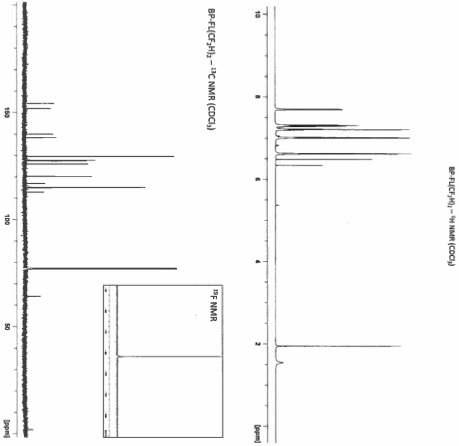


**Figure 31.** NMR spectra for Bisphenol FL (CF_2_H)_2_.


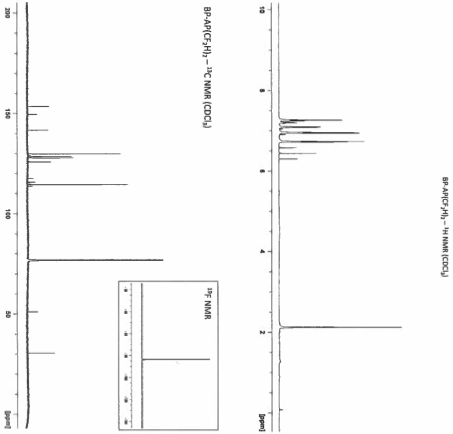


**Figure 32.** NMR spectra for Bisphenol AP (CF_2_H)_2_.


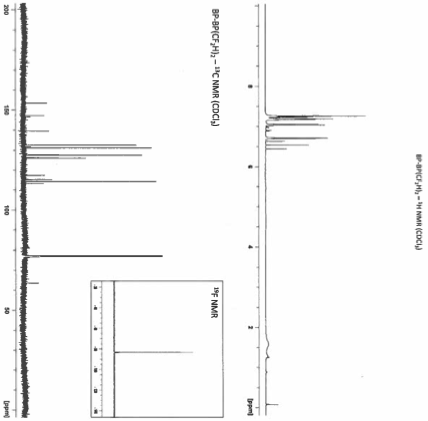


**Figure 33.** NMR spectra for Bisphenol BP (CF_2_H)_2_.


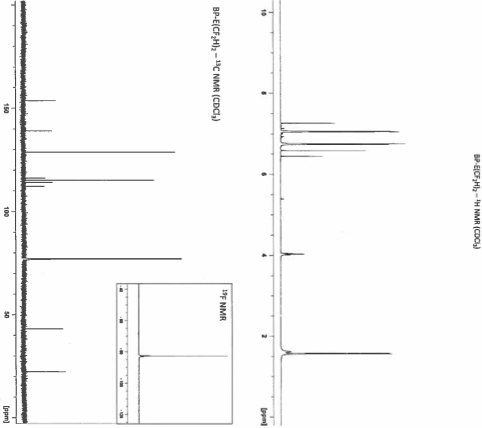


**Figure 34.** NMR spectra for Bisphenol E (CF_2_H)_2_.


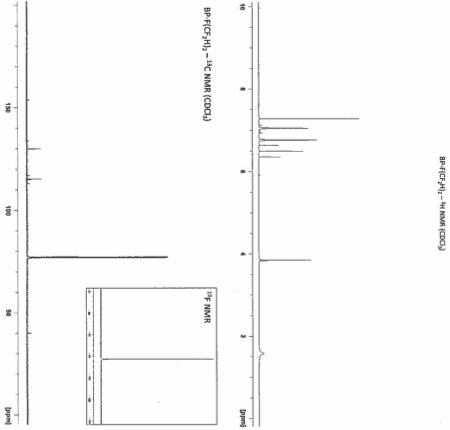


**Figure 35.** NMR spectra for Bisphenol F (CF_2_H)_2_.
